# Neuroblastoma patient outcomes, tumor differentiation, and ERK activation are correlated with expression levels of the ubiquitin ligase UBE4B

**DOI:** 10.18632/genesandcancer.97

**Published:** 2016-01

**Authors:** Sarah E. Woodfield, Rong Jun Guo, Yin Liu, Angela M. Major, Emporia Faith Hollingsworth, Sandra Indiviglio, Sarah B. Whittle, Qianxing Mo, Andrew J. Bean, Michael Ittmann, Dolores Lopez-Terrada, Peter E. Zage

**Affiliations:** ^1^ Department of Pediatrics, Section of Hematology/Oncology, Baylor College of Medicine, Houston, TX, USA; ^2^ Department of Pathology and Immunology, Baylor College of Medicine, Houston, TX, USA; ^3^ Department of Neurobiology and Anatomy, The University of Texas Medical School & Graduate School of Biomedical Sciences, Houston, TX, USA; ^4^ Department of Biomedical Engineering, University of Texas at Austin, Austin, TX, USA; ^5^ Department of Medicine, Dan L. Duncan Cancer Center, Baylor College of Medicine, Houston, TX, USA; ^6^ The Michael E. DeBakey Department of Veterans Affairs Medical Center, Houston, TX, USA; ^7^ Department of Pathology, University of Alabama Birmingham, Birmingham, AL, USA

**Keywords:** neuroblastoma, UBE4B, differentiation, ERK, retinoic acid

## Abstract

**Background:**

UBE4B is an E3/E4 ubiquitin ligase whose gene is located in chromosome 1p36.22. We analyzed the associations of UBE4B gene and protein expression with neuroblastoma patient outcomes and with tumor prognostic features and histology.

**Methods:**

We evaluated the association of *UBE4B* gene expression with neuroblastoma patient outcomes using the R2 Platform. We screened neuroblastoma tumor samples for UBE4B protein expression using immunohistochemistry. FISH for *UBE4B* and 1p36 deletion was performed on tumor samples. We then evaluated *UBE4B* expression for associations with prognostic factors and with levels of phosphorylated ERK in neuroblastoma tumors and cell lines.

**Results:**

Low *UBE4B* gene expression is associated with poor outcomes in patients with neuroblastoma and with worse outcomes in all patient subgroups. UBE4B protein expression was associated with neuroblastoma tumor differentiation, and decreased UBE4B protein levels were associated with high-risk features. UBE4B protein levels were also associated with levels of phosphorylated ERK.

**Conclusions:**

We have demonstrated associations between *UBE4B* gene expression and neuroblastoma patient outcomes and prognostic features. Reduced UBE4B protein expression in neuroblastoma tumors was associated with high-risk features, a lack of differentiation, and with ERK activation. These results suggest UBE4B may contribute to the poor prognosis of neuroblastoma tumors with 1p36 deletions and that UBE4B expression may mediate neuroblastoma differentiation.

## INTRODUCTION

Cases of high-risk neuroblastoma are associated with frequent relapses and tumors that are resistant to chemotherapy treatment, and children with refractory or recurrent neuroblastoma have poor responses to salvage therapy and very poor survival rates [1, 2]. A better understanding of the mechanisms of neuroblastoma tumorigenesis will likely provide improved treatment options for these children.

Deletions in chromosome 1p36 have been detected in approximately one-third of neuroblastoma tumors and are associated with high-risk tumor features and a poor prognosis [3-5]. UBE4B is an E3/E4 ubiquitin ligase whose gene is located at chromosome 1p36.22 [6, 7], and *UBE4B* gene expression has been previously shown to be reduced in a cohort of high-stage tumors compared to low-stage tumors [8, 9]. We have identified an association between *UBE4B* gene expression and neuroblastoma patient outcomes [10], suggesting a potential role for *UBE4B* as a tumor suppressor gene. The functional role of UBE4B in neuroblastoma pathogenesis and its association with other prognostic factors, however, are poorly understood.

We hypothesized that UBE4B gene and protein expression would be associated with neuroblastoma patient outcomes and other prognostic features and with neuroblastoma tumorigenesis. To explore the roles of UBE4B expression and function in the pathogenesis of neuroblastoma, we evaluated the association of *UBE4B* gene expression with neuroblastoma patient outcomes and prognostic features, and we investigated the expression of UBE4B protein in neuroblastoma tumor samples and its association with known prognostic features and downstream intracellular signaling pathways.

## RESULTS

### Association of *UBE4B* Gene Expression with Neuroblastoma Patient Outcomes

**Figure 1:**
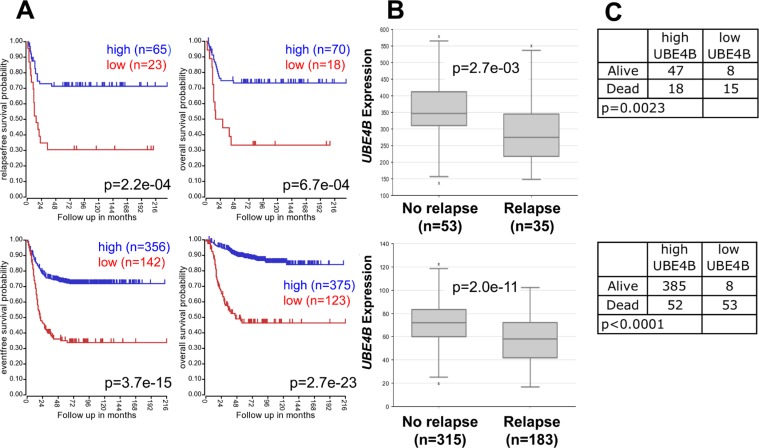
Neuroblastoma Patient Outcomes Based on *UBE4B* Expression **A.** Using the neuroblastoma Versteeg (top) and SEQC (bottom) patient data-sets in the R2 Genomics Analysis and Visualization Platform (http://r2.amc.nl), patients were divided into high (blue) and low (red) *UBE4B* gene expression groups by median-centered Log2 ratios and survival curves were generated. Relapse-free survival (top left), event-free survival (bottom left) and overall survival (right) curves are shown with patient numbers in parentheses. **B.** Relative *UBE4B* expression levels were plotted in patients with and without relapse from the Versteeg (top) and SEQC (bottom) patient data-sets, with patient numbers shown in parentheses. **C.** Numbers of patients with high or low *UBE4B* expression among patients either alive or dead at the time of data censoring for the Versteeg (top) and SEQC (bottom) data-sets.

We evaluated the association of *UBE4B* gene expression with neuroblastoma patient outcomes, using results from microarray analyses of neuroblastoma tumors obtained from the R2 Genomics Analysis and Visualization Platform. Low expression of *UBE4B* was significantly associated with reduced event-free, relapse-free, and overall survival rates in two large patient cohorts (Figure 1A). Furthermore, *UBE4B* gene expression was significantly lower among patients who experienced disease relapse, compared to those who did not have disease relapse (Figure 1B), and the percentage of deceased patients with low *UBE4B* expression in two separate datasets was 65.2% and 86.9%, respectively, compared to 27.7% and 11.9% among survivors (*p* = 0.0023 and *p* < 0.0001; Figure 1C). These results support the association of *UBE4B* gene expression with neuroblastoma patient outcomes, including an association between *UBE4B* gene expression and the risk of relapse and death.

### Association of *UBE4B* gene expression with neuroblastoma patient prognostic features

**Figure 2:**
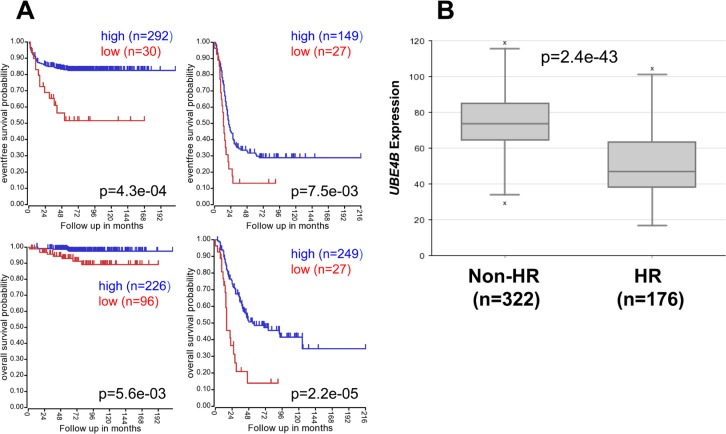

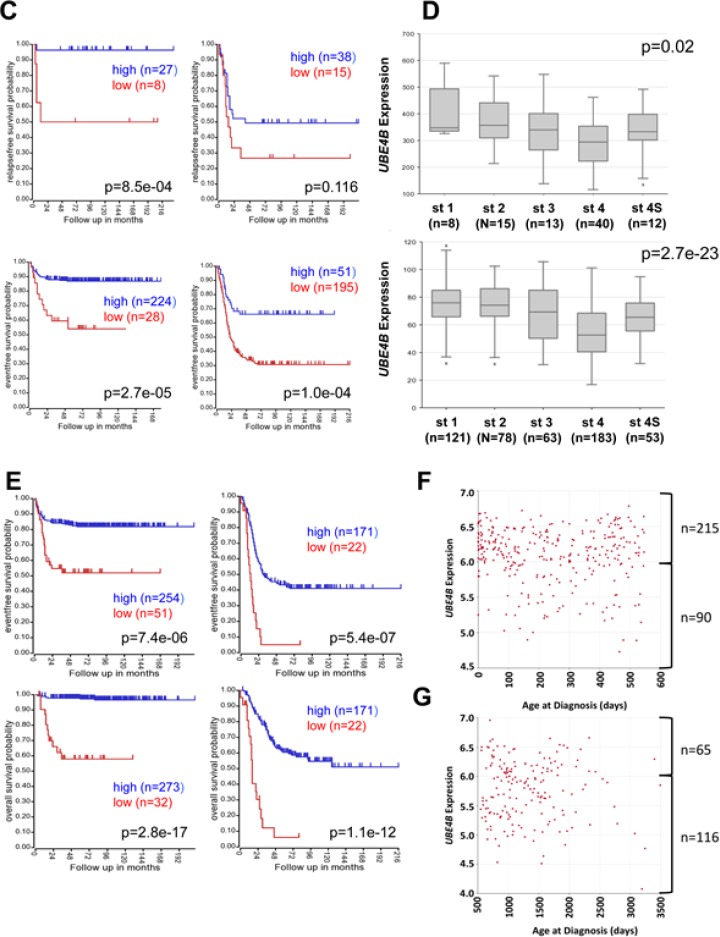
Neuroblastoma Patient Outcomes Based on *UBE4B* Expression and Known Neuroblastoma Prognostic Factors **A.** Using the neuroblastoma SEQC patient data-set in the R2 Genomics Analysis and Visualization Platform (http://r2.amc.nl), patients were divided into high (blue) and low (red) *UBE4B* gene expression groups by median-centered Log2 ratios and survival curves were generated for low and intermediate risk patients (left) and high risk patients (right). Event-free survival (top) and overall survival (bottom) curves are shown, with patient numbers in parentheses. **B.** Relative *UBE4B* expression levels were plotted in patients with non-high risk (Non-HR) and high-risk (HR) disease. **C.** Using the neuroblastoma Versteeg (top) and SEQC (bottom) patient data-sets, survival curves were generated for patients with stage 1, 2, and 4S tumors (left) and with stage 3 and 4 tumors (right). Relapse-free survival (top) and event-free survival (bottom) curves are shown, with patient numbers in parentheses. **D.** Relative *UBE4B* expression levels from the Versteeg (top) and SEQC (bottom) patient data-sets were plotted in patients with stage 1, 2, 3, 4, and 4S disease, respectively, with patient numbers shown in parentheses. **E.** Using the neuroblastoma SEQC patient data-set, survival curves were generated for patients <18 months of age at diagnosis (left) and patients >18 months of age at diagnosis (right). Event-free survival (top) and overall survival (bottom) curves are shown, with patient numbers in parentheses. (F.,G.) Relative UBE4B expression levels were plotted compared to the age of patients at diagnosis for patients ≤18 months of age at diagnosis (**F.)** and for patients >18 months of age at diagnosis (**G.)**.

To evaluate the potential association of *UBE4B* gene expression with known clinical and biological factors associated with neuroblastoma patient outcomes, we analyzed results from microarray analyses of neuroblastoma tumors. In neuroblastoma patient cohorts separated by treatment risk group, low *UBE4B* expression was associated with lower event-free and overall survival rates in patients with both low and intermediate risk neuroblastoma and in those with high-risk neuroblastoma (Figure 2A). *UBE4B* expression was also significantly lower in the cohort of patients with high-risk disease, compared to those with low and intermediate risk disease (*p* = 2.4e-43; Figure 2B).

In neuroblastoma patient cohorts separated by tumor stage, low *UBE4B* gene expression was again associated with lower relapse-free, event-free, and overall survival rates in patients with both low stage and high stage disease (Figure 2C, Supplemental Figure 1) and with stage 4 disease separately (Supplemental Figure 1). Furthermore, *UBE4B* gene expression was significantly lower in patients with stage 4 disease compared to other stages (Figure 2D). In patient cohorts separated by patient age, low *UBE4B* gene expression was also associated with lower relapse-free, event-free, and overall survival rates in patients diagnosed at both less than and greater than 18 months of age (Figure 2E, Supplemental Figure 2A), and *UBE4B* gene expression was inversely correlated with patient age at diagnosis (Significance of correlation: *r* = −0.327, *p* = 1.3e-13), with 215 out of 305 (70.5%) patients diagnosed at ≤18 months of age having high levels of *UBE4B* expression, compared to 65 out of 181 patients (35.9%) diagnosed at >18 months of age (*p* < 0.0001; Figure 2F, 2G). *UBE4B* expression was also significantly lower in a separate cohort of patients diagnosed at greater than 18 months of age, compared to those diagnosed at a younger age (Supplemental Figure 2B).

**Figure 3:**
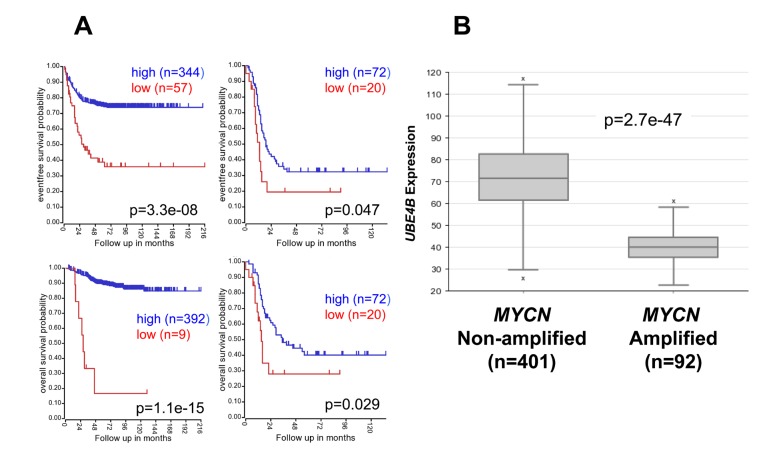
Neuroblastoma Patient Outcomes Based on *UBE4B* Expression and *MYCN* Amplification **A.** Using the neuroblastoma SEQC patient data-set in the R2 Genomics Analysis and Visualization Platform (http://r2.amc.nl), patients were divided into high (blue) and low (red) *UBE4B* gene expression groups by median-centered Log2 ratios and survival curves were generated for patients with non-*MYCN* amplified tumors (left) and with *MYCN* amplified tumors (right). Event-free survival (top) and overall survival (bottom) curves are shown, with patient numbers in parentheses. **B.** Relative *UBE4B* expression levels were plotted in patients with *MYCN* non-amplified and amplified tumors.

We have previously identified a potential association between low *UBE4B* gene expression and *MYCN* gene amplification [10]. To confirm the association between *UBE4B* expression and patient outcomes in patients with *MYCN*-amplified and non-amplified tumors, neuroblastoma patient cohorts were separated by the presence or absence of *MYCN* amplification. Patients with low *UBE4B* gene expression had lower rates of event-free and overall survival in cohorts with both *MYCN* amplified and non-amplified tumors (Figure 3, Supplemental Figure 3A), with the most significant effects observed in the *MYCN* non-amplified cohort. Furthermore, *UBE4B* gene expression was also significantly lower in patients with *MYCN* amplified tumors compared to non-amplified tumors (*p* = 2.7e-47; Figure 3, Supplemental Figure 3B). These results demonstrate the association of low *UBE4B* gene expression with poor outcomes in all neuroblastoma patient subsets separated by clinically relevant prognostic factors.

### Association of *UBE4B* gene expression with chromosome 1p36 deletion

**Figure 4:**
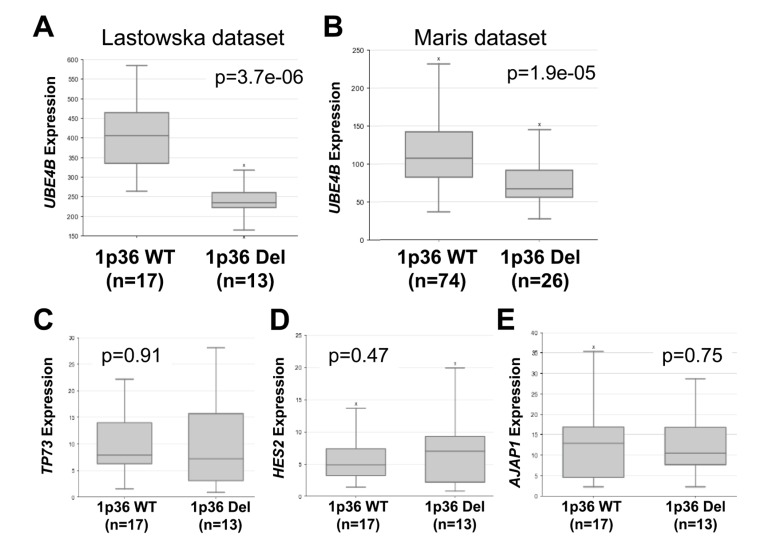
*UBE4B* Expression Compared to Chromosome 1p36 Status Using the R2 Genomics Analysis and Visualization Platform, *UBE4B* gene expression in the Lastowska (**A.)** and Maris (**B.)** datasets was compared in patients with wild-type chromosome 1p (left) or with 1p36 deletion (right), with patient numbers shown in parentheses. Expression of *TP73* (**C**.), *HES2* (**D.)**, and *AJAP1* (**E.)** in patients with wild-type chromosome 1p (left) or with 1p36 deletion (right) was compared in the Lastowska dataset.

The *UBE4B* gene is located within the chromosome 1p36 region frequently deleted in neuroblastoma tumors, and therefore *UBE4B* represents a candidate tumor suppressor gene within this region. In order to determine whether *UBE4B* gene expression was correlated with chromosome 1p36 deletion, we analyzed results from datasets in the R2 Genomics Analysis and Visualization Platform with information about tumor 1p36 status. In two separate datasets, *UBE4B* gene expression was significantly lower in the tumors from patients with chromosome 1p36 deletion, compared to those with wild-type chromosome 1p36, while the expression of three other genes located in the adjacent 1p36 region (*TP73*, *HES2*, and *AJAP1*) was unchanged (Figure 4), suggesting additional mechanisms of regulation of *UBE4B* gene expression are involved in neuroblastoma tumors.

To determine whether the *UBE4B* gene was lost in all cases of chromosome 1p36 deletion, institutional tumor samples were analyzed by FISH for both 1p36 status and the *UBE4B* gene. Out of 34 tumor samples, 7 were found to have chromosome 1p36 loss, and all 7 also had loss of the *UBE4B* gene. Neither 1p36 nor *UBE4B* loss was identified in any of the remaining 27 tumor samples (Supplemental Figure 4), suggesting that loss of *UBE4B* likely occurs in the majority of cases of chromosome 1p36 deletion.

### UBE4B protein expression is associated with neuroblastoma tumor cell differentiation

**Figure 5:**
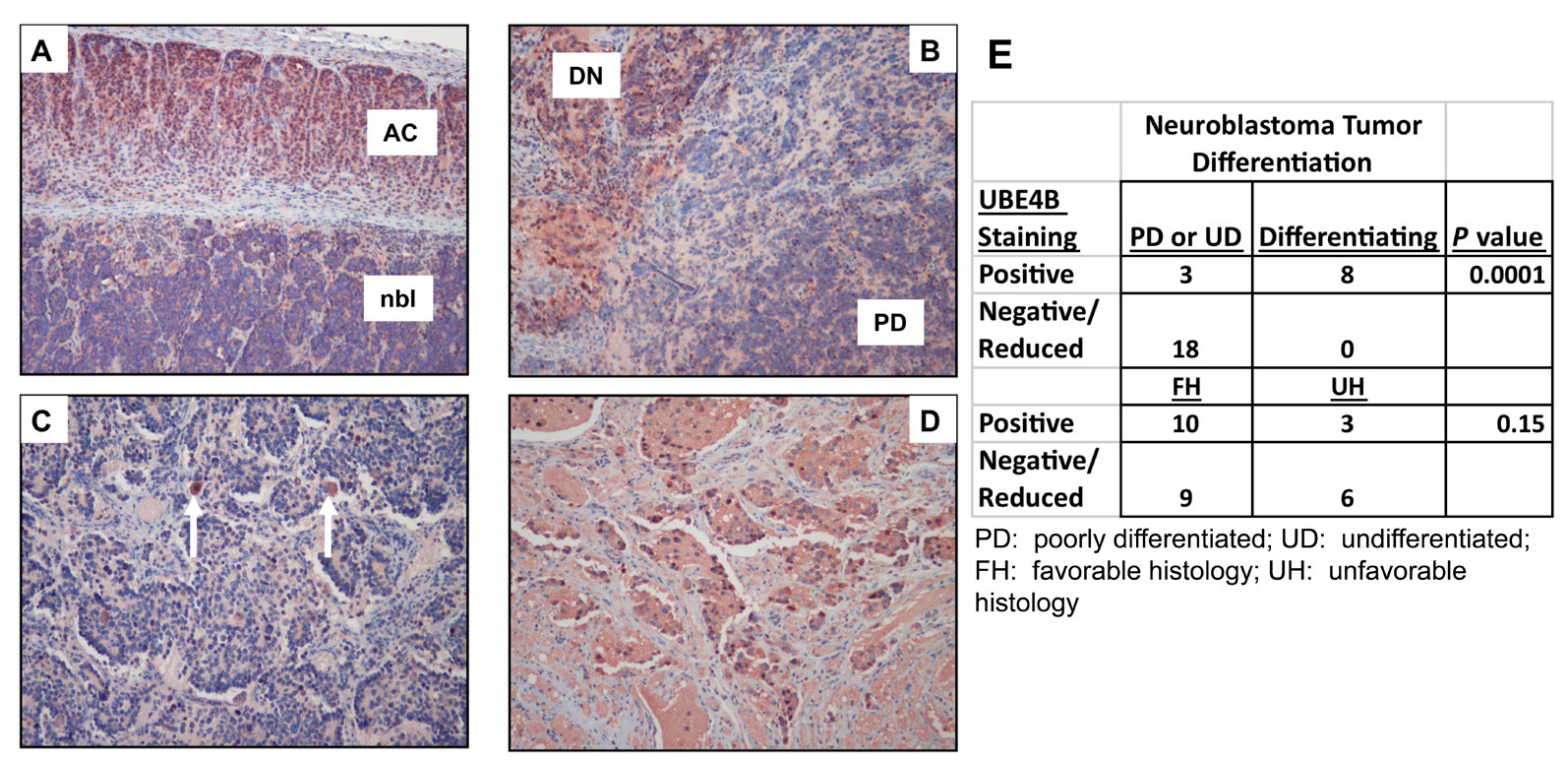
Reduced UBE4B Expression Is Associated with Poorly Differentiated Tumors **A.-D.** Formalin-fixed tumor samples were obtained from the Texas Children's Hospital tissue bank and sections were stained for the UBE4B protein by immunohistochemistry; **A.** Undifferentiated neuroblastoma (nbl) adjacent to normal adrenal cortex (AC); **B.** Poorly differentiated neuroblastoma nodule (PD, bottom right corner) adjacent to a differentiating neuroblastoma nodule (DN, upper left corner); **C.** Poorly differentiated neuroblastoma containing scattered ganglion-like cells (arrows); **D.** differentiating neuroblastoma tumor. **E.** UBE4B staining and differentiation status information from institutional tumor samples was compiled and compared.

To determine whether UBE4B protein expression was also associated with neuroblastoma patient outcomes and prognostic features, we analyzed neuroblastoma tumor samples for UBE4B expression by immunohistochemistry. In institutional tumor samples, reduction of UBE4B protein expression was only seen in poorly differentiated or undifferentiated neuroblastoma tumors or the poorly differentiated component of intermixed ganglioneuroblastomas, and UBE4B protein expression was significantly associated with neuroblastoma tumor differentiation in the cohort of samples (*p* < 0.0001; Figure 5).

**Figure 6:**
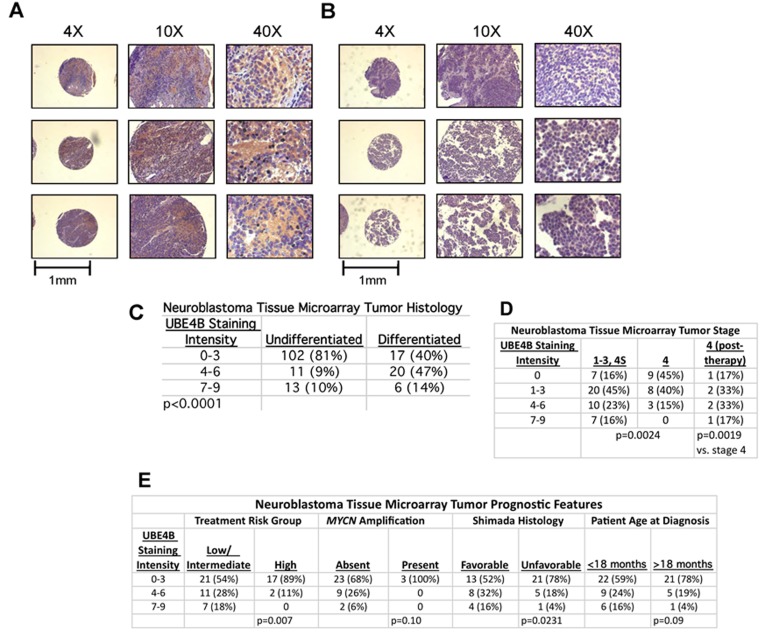
UBE4B Expression in Neuroblastoma Tumor Tissue Arrays Neuroblastoma tumor tissue microarrays (TMAs) were obtained from COG and were stained for UBE4B. Representative samples with positive staining (**A.)** and negative staining (**B.)** are shown. Scale bars are equal to 1 mm. (**C.)** UBE4B staining score was determined in blinded fashion and compared to tumor differentiation in each sample. UBE4B staining score was also compared to tumor stage (**D.)** and other prognostic features (**E.)**.

To further analyze the association of UBE4B protein with neuroblastoma tumor differentiation, neuroblastoma tissue microarrays (TMAs) obtained from the Children's Oncology Group (COG) (Supplemental Figure 5) were analyzed by immunohistochemistry for UBE4B protein expression and tumor differentiation. In TMA samples, 26 of 43 differentiated tumor samples (61%) demonstrated increased UBE4B protein expression, compared to 24 out of 126 (19%) undifferentiated tumor samples (*p* < 0.0001; Figure 6A-6C).

In order to evaluate whether UBE4B protein expression was correlated with any other known neuroblastoma prognostic factors, UBE4B staining intensity was compared in patient cohorts divided by tumor stage, treatment risk group, *MYCN* amplification, Shimada histology, and patient age at diagnosis. 37 out of 44 low stage neuroblastoma tumors (84%) demonstrated UBE4B protein expression, including 9 out of 11 patients with stage 4S neuroblastoma (82%). By comparison, only 11 of 20 (55%) untreated stage 4 tumors had UBE4B protein expression, with 9 out of 20 (45%) having absent UBE4B expression (*p* = 0.0024; Figure 6D). In samples obtained from patients with stage 4 disease obtained after chemotherapy treatment, 5 of 6 patients demonstrated increased UBE4B expression (83%; *p* = 0.0019), suggesting that an increase in *UBE4B* expression occurs in response to chemotherapy treatment in patients with stage 4 neuroblastoma.

Further analysis also demonstrated that low UBE4B protein expression was significantly associated with high risk disease and unfavorable Shimada histology. Increased UBE4B protein expression was also seen in tumors with non-amplified *MYCN* and in tumors from patients diagnosed at over 18 months of age, but these trends did not reach statistical significance (Figure 6E). In samples with available information about patient outcomes, we observed that low UBE4B protein expression was associated with a non-significant trend towards decreased survival (Supplemental Figure 6), further suggesting that the association of *UBE4B* gene expression with neuroblastoma patient outcomes is mediated by UBE4B protein expression and function.

### *UBE4B* expression in neuroblastoma tumor cells is increased after retinoic acid treatment and is associated with RAS/MAPK pathway activity

To evaluate changes in *UBE4B* gene expression during neuroblastoma tumor cell differentiation, we performed RNA-sequencing on neuroblastoma tumor cells before and after 13-cis-retinoic acid (CRA) treatment. In SK-N-BE(2), SK-N-SH, and IMR-32 neuroblastoma tumor cell lines tested after 48 hours of retinoic acid exposure, *UBE4B* gene expression increased by an average of 23.1% (+/− 19.6%)(Supplemental Figure 7).

**Figure 7:**
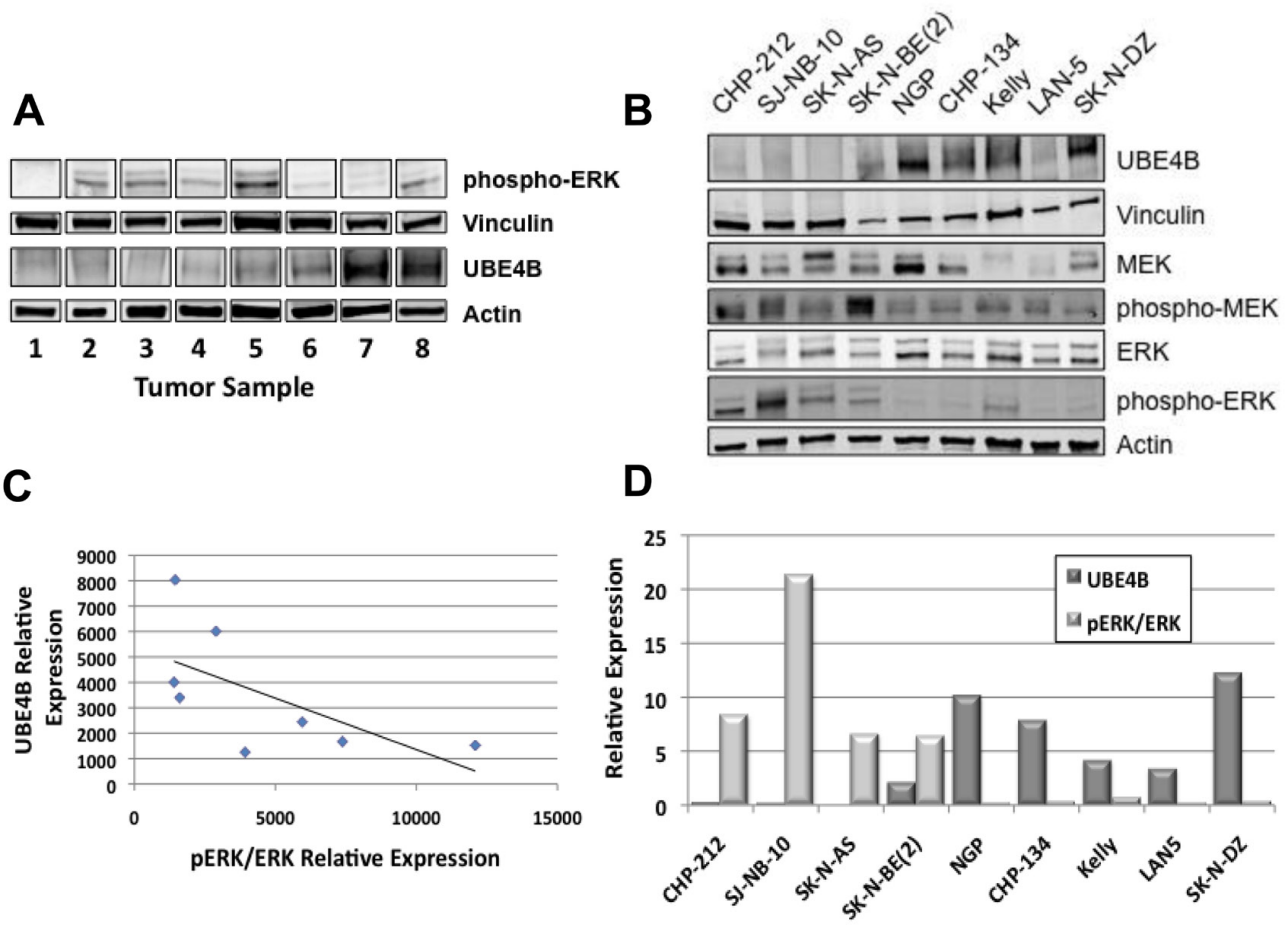
UBE4B, MEK, and ERK Expression in Neuroblastoma Tumor Cell Lines and Tissue Samples (**A**.) Neuroblastoma patient samples were lysed and Western blots for UBE4B and phosphorylated ERK were performed. Vinculin and actin were used as loading controls. (**B.)** A panel of neuroblastoma cell lines were lysed and Western blots for UBE4B and for total and phosphorylated MEK and ERK were performed. Vinculin and actin was used as loading controls. Densitometry analysis was performed to quantify relative phospho-ERK and UBE4B protein levels in neuroblastoma tumor samples (**C.)** and cell lines (**D.)** Spearman's correlation analysis was performed with UBE4B and pERK protein levels as shown. R = −0.7381 and *p* = 0.03655 for tumor samples (**C.)** and R = −0.78333 and *p* = 0.01252 for cell lines **(D.)**.

ERK activation has been shown to correlate with nerve growth factor-induced neuroblastoma cell differentiation [11], suggesting a role for the RAS/MAPK pathway in neuroblastoma differentiation. In order to determine whether UBE4B expression was also associated with RAS/MAPK pathway signaling activity in neuroblastoma tumor cells, we determined levels of UBE4B and total and phosphorylated MEK and ERK expression in established neuroblastoma cell lines and patient tumor samples. Neuroblastoma cell lines and tumors demonstrated varying levels of phosphorylated MEK and ERK. In both cell lines and patient tumor samples, decreased UBE4B expression was associated with the highest levels of phosphorylated ERK, while cell lines with increased UBE4B had lower levels of phosphorylated ERK (Figure 7), suggesting an inverse association between UBE4B levels and RAS/MAPK pathway activity and providing a mechanistic link between UBE4B protein expression and neuroblastoma tumor differentiation.

## DISCUSSION

Children with high-risk neuroblastoma have extremely poor outcomes, and additional understanding of the pathways involved in neuroblastoma pathogenesis will assist in the development of improved therapies. The *UBE4B* gene is located in chromosome 1p36.22, a commonly deleted chromosomal region in neuroblastoma tumors, and our results demonstrate an association of UBE4B gene and protein expression with neuroblastoma patient outcomes and prognostic features and of UBE4B protein levels with neuroblastoma tumor cell differentiation, suggesting a role for *UBE4B* as a novel tumor suppressor gene.

We previously identified an interaction between UBE4B and the endosomal membrane protein hepatocyte growth factor-regulated tyrosine kinase substrate (Hrs), and we have demonstrated that the UBE4B-Hrs interaction is critical for appropriate growth factor receptor GFR trafficking and degradation [7, 10, 12], suggesting a previously undiscovered link between GFR trafficking and neuroblastoma pathogenesis. Recent studies have identified a role for TrkA ubiquitination by the ubiquitin ligase Cbl-b in neuroblastoma differentiation [11], further suggesting a link between receptor trafficking and neuroblastoma pathogenesis.

We have identified an association between UBE4B protein expression and poor prognostic features for children with neuroblastoma, although we were unable to demonstrate a significant association between UBE4B protein expression and patient outcomes. These analyses were limited by the small number of patients with outcomes data available and the limited number of events among those patients. Further analyses using larger patient cohorts will be needed to confirm the potential association between protein expression and patient survival. Further studies are also needed to confirm whether UBE4B protein function is also linked to patient prognosis and whether UBE4B plays a functional role in neuroblastoma tumor differentiation.

Although we have demonstrated associations between UBE4B gene and protein expression and a number of critical neuroblastoma prognostic variables, studies are ongoing to determine whether UBE4B expression is independently associated with neuroblastoma patient outcomes. We have shown that neuroblastoma tumors with 1p36 deletion have lower *UBE4B* expression levels, suggesting that decreased *UBE4B* gene expression is a potential contributor to the poor prognosis of neuroblastoma patients with 1p36 deletions. However, a large number of genes located within the 1p36 region have been proposed as tumor suppressors in neuroblastoma and other tumors, including *CHD5* [13], *CAMTA1* [14, 15], miRNA-34a [16, 17], *CASZ1* [18], *KIF1B* [19, 20], and the *HES* gene family [21]. Low expression levels of many of these genes have been correlated with poor patient outcomes, suggesting that 1p36 deletion may impact prognosis *via* effects on multiple independent pathways. However, the lack of effect of 1p36 deletion on the expression of all genes in this region suggests that other mechanisms of gene regulation are involved. We were unable to find any tumors where *UBE4B* was lost in the absence of 1p36 deletion. However, the number of patient samples screened was small and does not preclude cases of 1p36 deletion without loss of *UBE4B*, or of *UBE4B* loss in the absence of 1p36 deletion. Furthermore, the association of low *UBE4B* expression with worse patient outcomes in all patient subsets suggests that alternate mechanisms regulating *UBE4B* gene expression other than 1p36 deletion are likely involved in neuroblastoma tumors. Further studies are underway to identify whether other genetic or epigenetic events are responsible for regulation of *UBE4B* gene expression.

The significant association of low UBE4B expression with lower survival rates in cohorts of low stage and younger patients suggests UBE4B may be a novel predictor of relapse in children with low and intermediate risk neuroblastoma. Prior studies have identified chromosome 1p loss of heterozygosity (LOH) as a prognostic indicator for disease recurrence in children with localized neuroblastoma [22], and have also determined that alternations in both chromosome 1p and 11q are poor prognostic factors in patients with low stage neuroblastoma [23]. Prior studies have also identified gene expression profiles able to differentiate infant stage 4 from stage 4S neuroblastoma [24]. For these infants with metastatic disease, UBE4B protein expression may serve as a novel molecular marker to distinguish stage 4 from 4S disease, and UBE4B expression and function may underlie the dramatic clinical and biologic differences between these two subtypes of neuroblastoma.

The role of RAS/MAPK pathway signaling in neuroblastoma tumor cells is poorly understood. Activating mutations in the genes of members of the RAS/MAPK pathway have been identified in a small subset of neuroblastoma tumors [25] at diagnosis and more frequently at relapse [26]. Furthermore, expression of the gene for the Ras GTPase-activating protein (RasGAP) NF1 is also associated with neuroblastoma patient outcomes [27, 28], suggesting a more significant role for the RAS/MAPK pathway in neuroblastoma pathogenesis and disease relapse.

The mechanisms underlying neuroblastoma tumor cell differentiation are poorly understood. A number of proteins have been shown to be required for or involved in the effects of CRA on neuroblastoma tumor cells, including RET (29,30), REST (31), and ZNF423 (32). The expression of the Chromodomain-helicase DNA binding protein 5 (*CHD5*) gene was recently shown to be elevated after 7 days of CRA treatment in SH-SY5Y, NGP, and SK-N-DZ neuroblastoma cells (33), although the increased expression was seen after the induction of morphologic neuroblastoma differentiation. The relative role of UBE4B and other proteins in the induction of differentiation remain unclear, and further studies are underway to further delineate the mechanisms underlying this process.

We have confirmed an association between UBE4B expression and neuroblastoma patient outcomes and prognostic features and demonstrated that reduced UBE4B expression in neuroblastoma tumors was associated with a lack of differentiation. This link between a known cytogenetic risk factor, GFR trafficking, and neuroblastoma tumor histology suggests UBE4B-mediated GFR trafficking may contribute to the poor prognosis of neuroblastoma tumors with 1p36 deletions. UBE4B therefore represents a novel molecular marker of neuroblastoma tumor differentiation and intratumoral heterogeneity and also represents a potential target for novel drug development.

## MATERIALS AND METHODS

### Neuroblastoma cell lines and culture conditions

The neuroblastoma cell lines used in this study have been previously described [34-41] and were generously provided by Shahab Asgharzadeh (Children's Hospital Los Angeles, Los Angeles, CA), Susan Cohn (The University of Chicago Children's Hospital, Chicago, IL), Jill Lahti (St. Jude Children's Research Hospital, Memphis, TN), John Maris (Children's Hospital of Philadelphia, Philadelphia, PA), William Weiss (The University of California, San Francisco, San Francisco, CA) or were purchased from the American Type Culture Collection (ATCC; Rockville, MD). Cell lines were grown at 37° in 5% CO2 in appropriate media (Invitrogen, Carlsbad, CA) supplemented with 10% heat-inactivated fetal bovine serum (FBS) (Life Technologies, Grand Island, NY), L-glutamine, sodium pyruvate, and non-essential amino acids as previously described [42]. All cell lines were authenticated by DNA profiling prior to use.

### Neuroblastoma patient tumor samples and data

Representative formalin-fixed, paraffin embedded (FFPE) tumor tissue blocks were selected for the study following histological case review (RJG and DLT) and retrieved from the archives of the Texas Children's Hospital Department of Pathology. Fresh frozen patient tumor samples were obtained from the Texas Children's Hospital Research Tissue Support Services (RTSS). Fresh resected neuroblastoma tumor samples were collected from patients after informed consent was obtained *via* an Institutional Review Board-approved tissue banking protocol. Samples were placed in sterile human stem cell media and flash frozen in liquid nitrogen for storage by RTSS. Neuroblastoma patient tumor tissue microarrays (TMAs) were provided by the Children's Oncology Group Neuroblastoma Biology Committee and the Biopathology Center in Columbus, OH [10]. We obtained microarray analysis results of neuroblastoma patient tumor samples from the R2 Genomics Analysis and Visualization Platform (http://r2.amc.nl) using the Versteeg, SEQC, Lastowska, and Maris databases, which included comprehensive information on the relevant clinical and prognostic factors selected for analysis. *UBE4B* probesets in each database with the highest average signals (Versteeg probeset 202317_s_at; SEQC probeset NM_006048; Lastowska probeset 202317_s_at; Maris probeset 41339_at) were selected for analysis. All experiments utilizing patient tissue samples and analyzing patient information were approved by the Baylor College of Medicine Institutional Review Board.

### Immunohistochemistry

Formalin-fixed paraffin embedded (FFPE) tissue specimens from 35 archival neuroblastoma cases diagnosed at Texas Children's Hospital were used for immunohistochemical (IHC) stains. After heat-induced antigen retrieval in Target Retrieval Solution following the manufacturer's instructions (S1699; Dako, Carpinteria, CA), IHC staining was performed using the ImmPRESS HRP rabbit polyclonal detection kit (Vector Laboratories; Burlingame, CA) with a polyclonal antibody to UBE4B (ab97697; 1:400; Abcam, Cambridge, MA). Anaplastic medulloblastoma tissue samples were used as positive controls for UBE4B staining [43] in addition to internal control cells, such as adjacent adrenal cortical epithelial cells or ganglion cells. Slides were de-identified and randomly distributed to two diagnostic pathologists (RJG and DLT) for evaluation. Immunoreactivity was categorized as negative/reduced or positive. “Negative/reduced” was defined as reduced or absent UBE4B staining intensity in at least 50% of the tumor cells compared to positive external and internal controls. “Positive” was defined as UBE4B staining intensity similar to or stronger than positive external and internal controls in at least 50% of the tumor cells. Neuroblastoma tumor differentiation was determined using standard criteria as previously described [44]. Photographs of stained tumor samples were taken using an Olympus DP71 camera attached to a Nikon Eclipse E100 microscope at 20x magnification.

For immunohistochemical staining of TMA tumor samples, TMA slides were deparaffinized and hydrated. Slides were boiled in Target Retrieval Solution (Dako) and blocked with 10% normal serum with 1% BSA in TBS. UBE4B primary antibody (ab97697, Abcam) was diluted 1:400 in SignalStain Antibody Diluent (Cell Signaling, Danvers, MA), and TMAs were incubated with UBE4B antibodies for 30 minutes at room temperature. UBE4B signal was detected using SignalStain Boost Detection Reagent (Cell Signaling) and SignalStain DAB Chromogen Concentrate (Cell Signaling). Slides were then counterstained with CAT hematoxylin (Biocare Medical, Concord, CA) and bluing reagent (VWR, Radnor, PA). Finally, slides were dehydrated and coverslips were mounted with Cytoseal XYL (Thermo Fisher Scientific, Waltham, MA). Slides were visualized using an inverted microscope (Nikon Eclipse TE-300, Nikon, Tokyo, Japan) and images were acquired on an RS Photometrics CoolSNAP color digital camera (Roper Scientific, Photometrics, Tucson, AZ) using RS Photometrics Image Software Version 1.9.2 (Roper Scientific, Photometrics, Tucson, AZ).

Tissue microarrays stained as above with antibodies to UBE4B were scored for both intensity of expression (scored 0-3) and extent of staining (scored 0-3) to give a 10 point multiplicative staining index of 0-9 as described previously [45] by a pathologist (MI) blind to patient identities, features, and outcomes.

### Fluorescence *in situ* hybridization (FISH)

We used institutional FFPE tissue specimens described above for FISH analyses. To detect the *UBE4B* gene copy number in neuroblastoma tumor samples, two BAC clones targeting the UBE4B gene, CTD-2254N9 and CTD-2171J21 (Life Technologies), were developed using a nick-translation kit (Abbott Molecular, Abbott Park, IL). FISH analyses for the centromere of chromosome 1, chromosome 1q25, and for chromosome 1p36 deletion were performed using a commercially available probes (CEP1, Vysis 1p36/1q25 Probe Kit; Abbott Molecular).

### Western blots

To measure the levels of proteins in patient samples, samples were homogenized on ice, then incubated for 30 minutes in radioimmunoprecipitation assay (RIPA) protein lysis buffer containing protease inhibitors (Sigma) and phosphatase inhibitors (Roche, San Francisco, CA) with homogenization every 10 minutes. Lysates were centrifuged and supernatants were collected. For protein levels in neuroblastoma cell lines, cells were plated in 100-mm plates and allowed to adhere and proliferate for two days with media being replaced with fresh media after one day. Cells from plates at approximately 80% confluency were then harvested and lysed as above.

Protein concentration in each sample lysate was measured using a protein assay dye reagent (Bio-Rad, Hercules, CA). 30-50 μg total denatured protein from each cell line or tumor sample lysate was separated by sodium dodecyl sulfate-polyacrylamide gel electrophoresis (SDS-PAGE) and transferred to nitrocellulose (Invitrogen) or polyvinylidene fluoride (PVDF) membranes using standard techniques. Membranes were blocked in Odyssey blocking buffer (Li-Cor, Lincoln, NE) for two hours at room temperature and then incubated overnight with primary antibodies to total MEK (9126; 1:1000; Cell Signaling), phosphorylated MEK (9154; 1:1000; Cell Signaling), total ERK (4695; 1:1000; Cell Signaling), phosphorylated ERK (4370; 1:2000; Cell Signaling), UBE4B (ab97697; 1:1000; Abcam), Actin (A5316 or A5441; 1:5000; Sigma), or Vinculin (1:10000; ab1290002; Abcam). Bound primary antibodies were incubated for two hours at room temperature with IRDye800 conjugated affinity purified anti-rabbit or anti-mouse secondary antibodies (1:5000; Rockland, Gilbertsville, PA), and the signal was visualized using an Odyssey infrared imaging system (Li-Cor).

### RNA-sequencing

SK-N-BE(2), SK-N-SH, and IMR-32 neuroblastoma tumor cells were seeded at 60-70% confluence and allowed to attach for 24 hours, with four plates per cell line. Cells in two plates for each cell line were treated with 5 μM 13-*cis*-retinoic acid for 48 hours, while other plates were left untreated. Cells were washed with PBS and lysed in the plates using Qiagen lysis buffer (Qiagen, Valencia, CA), and cell lysates from individual plates were homogenized separately using QIAshredder (Qiagen). Total cellular RNA was harvested from each lysate separately using the Qiagen mini RNeasy kit (Qiagen), following the manufacturer's instructions. RNA sample quality was confirmed using a NanoDrop 2000 spectrophotometer (Thermo Scientific, Wilmington, DE) and Agilent Bioanalyzer 2100 (Agilent Technologies, Santa Clara, CA). A double-stranded DNA library was created using the Illumina TruSeq RNA library preparation protocol. 250ng of total RNA, quantitated by picogreen, was used for each sample. cDNA was created using the fragmented 3′ poly (A) selected portion of total RNA and random primers. Libraries were created from the cDNA by first blunt ending the fragments, attaching an adenosine to the 3′ end and finally ligating unique adapters to the ends. The ligated products were then amplified using 15 cycles of PCR. The resulting libraries were quantitated using the NanoDrop spectrophotometer and fragment size assessed with the Agilent 2100 Bioanalyzer. A qPCR assay was performed on the libraries to determine the concentration of adapter ligated fragments using the Applied Biosystems ViiA 7 Quantitative PCR instrument and a KAPA Library Quant Kit. All samples were pooled equimolar and re- quantitated by qPCR, and re-assessed on the Bioanalyzer. Using the pooled concentration from the qPCR assay, the library pool was loaded onto each of two high output mode lanes at a concentration of 15pM, for cluster generation on the cBot and sequencing on the HiSeq 2500 (Illumina, San Diego, CA) at a read length of 100bp, using paired-end v3 chemistry.

### Statistical analysis

All microarray data generated from the R2 Genomics Analysis and Visualization Platform was analyzed using the R2 program for analysis and visualization of microarray data (http://r2.amc.nl). Kaplan-Meier analyses and comparisons of *UBE4B* expression between different patient subgroups were performed online and the resulting survival curves, box plots, and p values (obtained *via* the log-rank test) were downloaded as previously described [46]. Comparisons between UBE4B staining intensity and clinical or biologic variables were made using Chi-squared or Fisher-Exact tests, as appropriate.

Outcomes for patients for neuroblastoma TMA samples were obtained from COG. Patients were divided into high and low UBE4B protein expression groups according to their average multiplicative staining scores on TMA slides. The high-expression group contained 12 patients and the low-expression group contained 7 patients. Kaplan-Meier survival curves were plotted using the open-source statistical packages in R (http://www.r-project.org). Survival curves were compared by UBE4B protein expression groups using the log-rank test to examine the association between UBE4B expression and patient survival outcomes in TMA samples.

For RNA-sequencing analysis, 101-nucleotide reads were trimmed to 90 nucleotides by removing the low-quality nucleotides from the 5′ and 3′ ends of the reads. The resulting 90-nucleotide pair-ended reads were mapped to the human genome (UCSC hg19) using Tophat [47] with NCBI RefSeq genes as the reference. In order to reduce possible PCR biases, we removed the read duplicates using picard tools (http://broadinstitute.github.io/picard/). HTseq (http://www-huber.embl.de/users/anders/HTSeq) was used to determine the number of reads falling in the known genes. EdgeR [48] and DESeq2 [49] were used to analyze the gene-based read counts to detect differentially expressed genes between the groups of interest. The false discovery rate (FDR) of the differentially expressed genes was estimated using Benjamini and Hochberg method [50]. FDR < 0.05 was considered statistically significant.

## SUPPLEMENTARY INFORMATION FIGURES



## ABBREVIATIONS

TMA: tissue microarray
GFR: growth factor receptor
FISH: Fluorescence *in situ* hybridization
Hrs: hepatocyte growth factor-regulated tyrosine kinase substrate
FFPE: formalin fixed, paraffin-embedded
IHC: immunohistochemistry
MAPK: mitogen-activated protein kinase
MEK: MAPK/ERK kinase
ERK: extracellular signal-regulated kinase
RTSS: Research Tissue Support Service
