# Supplementary material for: Neuroblastoma patient outcomes, tumor differentiation, and ERK activation are correlated with expression levels of the ubiquitin ligase UBE4B
Source: Genes Cancer. 2016 Jan;7(1-2):13–26. doi: 10.18632/genesandcancer.97 (PMC4773702; doi:10.18632/genesandcancer.97)
Supplement: Supplementary file 1 [file ganc-07-013-s001.pdf]

Neuroblastoma patient outcomes, tumor differentiation, and ERK activation are correlated with expression levels of the ubiquitin ligase UBE4B – Woodfield et al

Supplementary Information

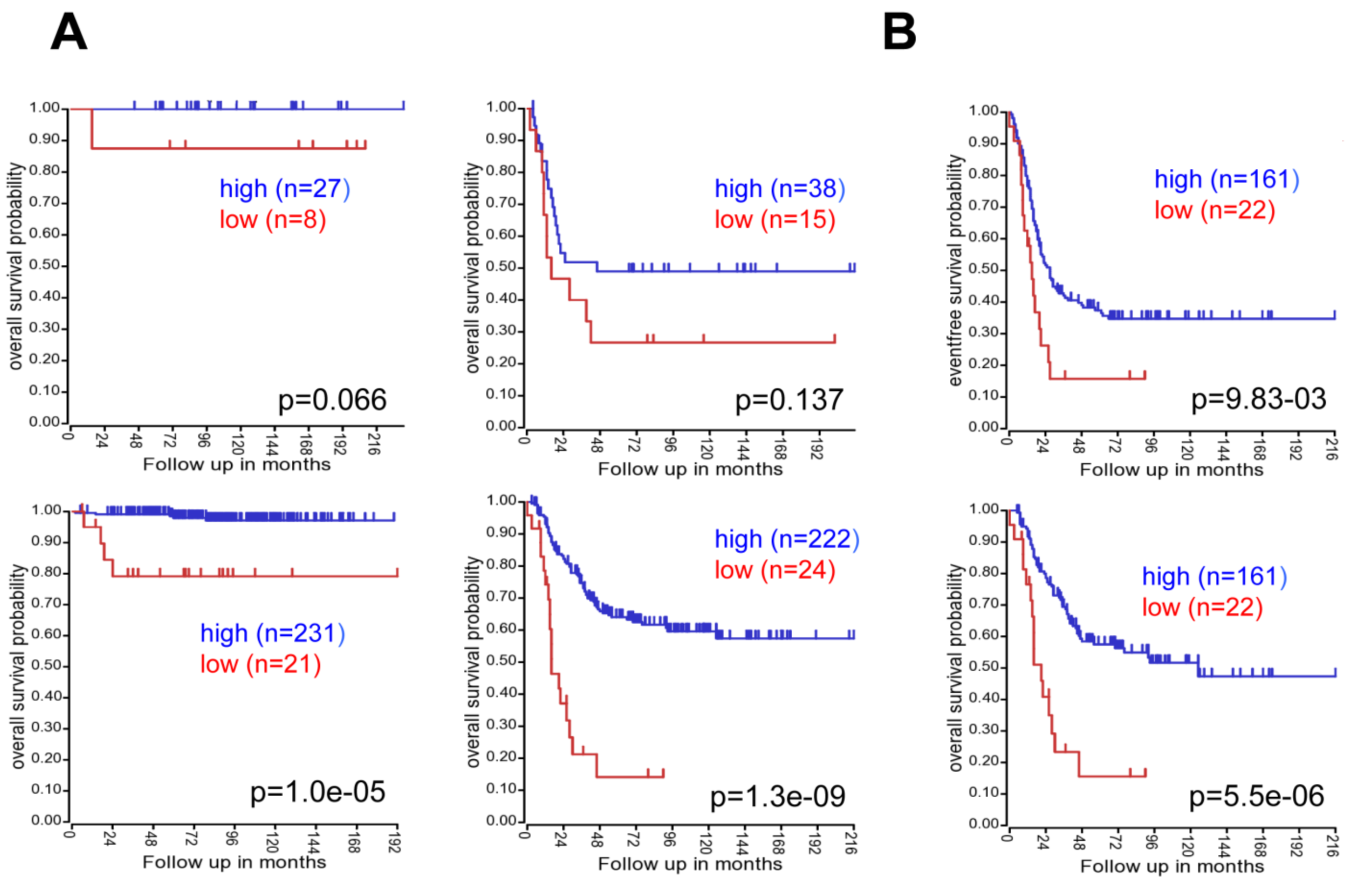

Supplemental Figure 1: Neuroblastoma Patient Subgroup Outcomes Based on UBE4B Expression and Known Prognostic Factors. (A) Using the Versteeg (top) and SEQC (bottom) neuroblastoma patient data-sets, overall survival curves were generated for patients with stage 1, 2, and 4S tumors (left) and with stage 3 and 4 tumors (right), with patient numbers in parentheses. (B) Using the SEQC dataset, patients were divided into high (blue) and low (red) *UBE4B* gene expression groups by median-centered Log2 ratios and Kaplan Meier curves were generated. Estimated event-free survival (top) and overall survival (bottom) curves were generated for neuroblastoma with stage 4 tumors.

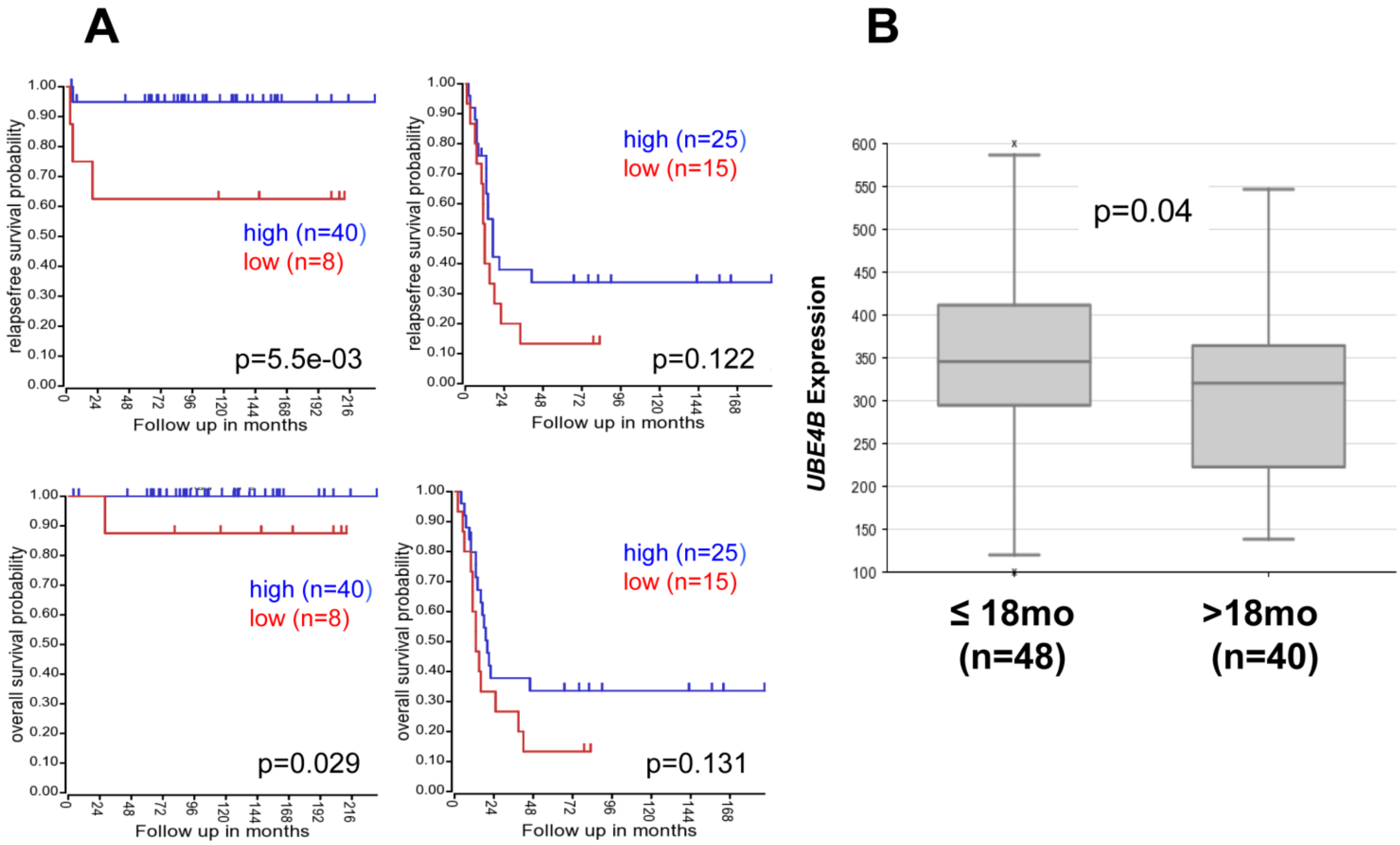

Supplemental Figure 2: Neuroblastoma Patient Outcomes Based on UBE4B Expression and Known Neuroblastoma Prognostic Factors. (A) Using the neuroblastoma Versteeg patient data-set in the R2 Genomics Analysis and Visualization Platform (http://r2.amc.nl), patients were divided into patients <18 months of age at diagnosis (left) and patients >18 months of age at diagnosis (right). Relapse-free survival (top) and overall survival (bottom) curves are shown, with patient numbers in parentheses. (B) Relative *UBE4B* expression levels were plotted compared to the age of patients at diagnosis

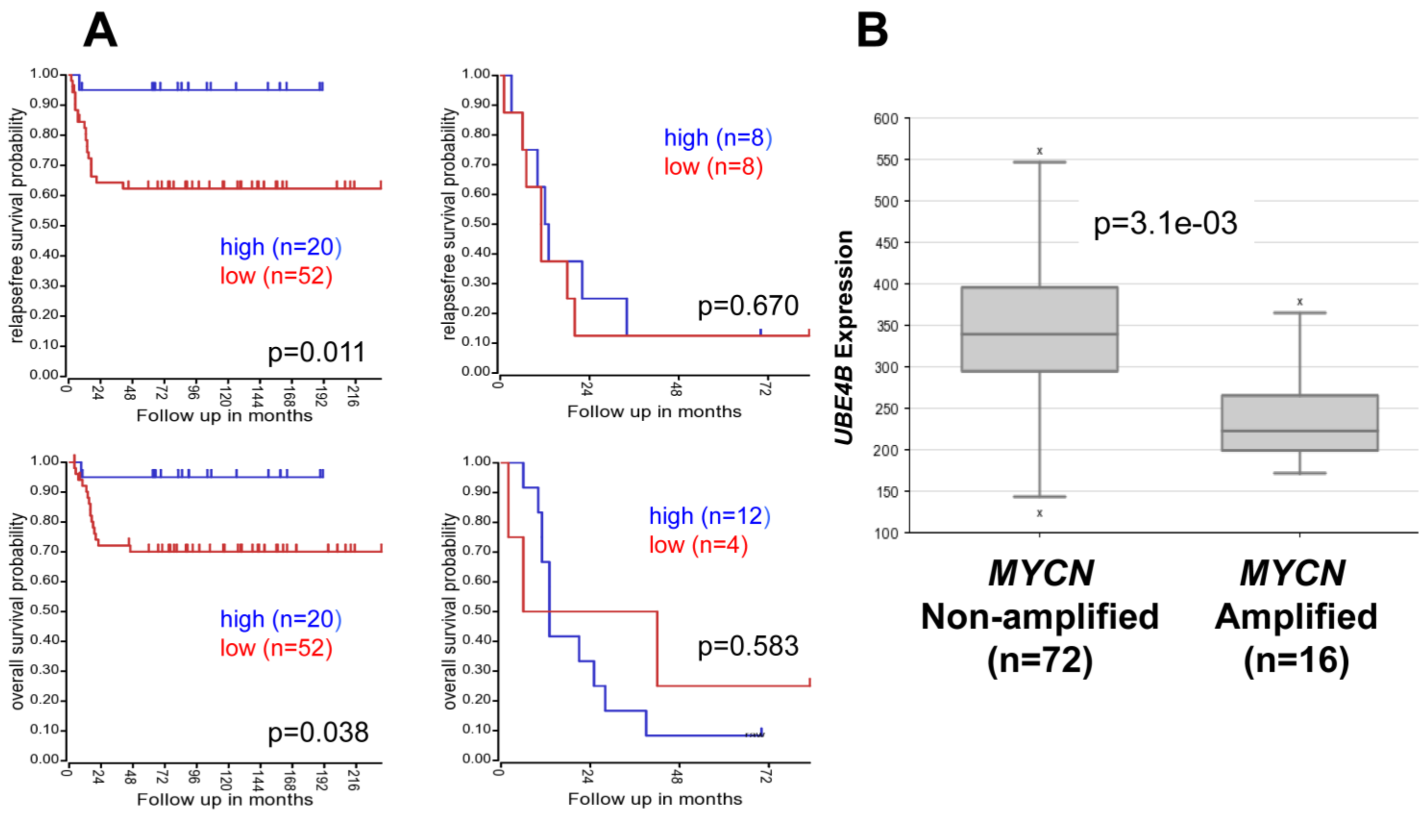

Supplemental Figure 3: Neuroblastoma Patient Outcomes Based on UBE4B Expression and MYCN Amplification. (A) Using the neuroblastoma Versteeg patient data-set in the R2 Genomics Analysis and Visualization Platform (http://r2.amc.nl), patients were divided into high (blue) and low (red) *UBE4B* gene expression groups by median-centered Log2 ratios and survival curves were generated for patients with non-*MYCN* amplified tumors (left) and with *MYCN* amplified tumors (right). Relapse-free survival (top) and overall survival (bottom) curves are shown, with patient numbers in parentheses. (B) Relative *UBE4B* expression levels were plotted in patients with *MYCN* non-amplified and amplified tumors.

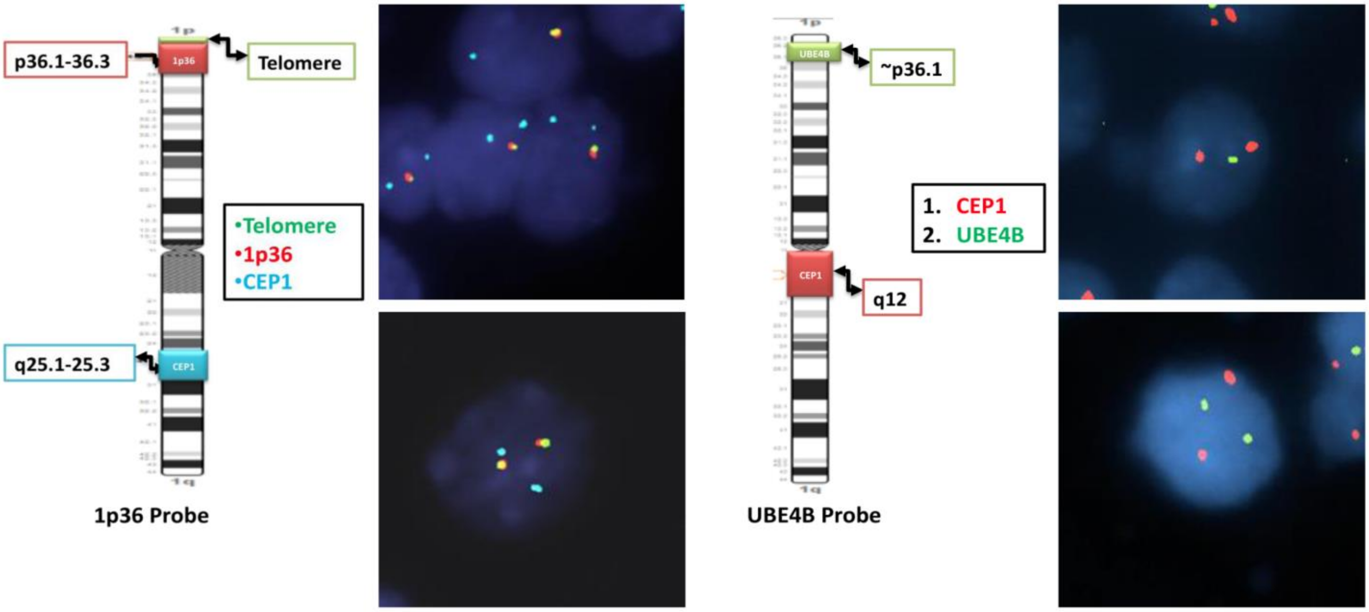

	UBE4B WT	UBE4B deletion
1p36 WT	27	0
1p36 deletion	0	7

Supplemental Figure 4: FISH Analysis of Neuroblastoma Tumor Samples for 1p36 and UBE4B deletion. Tumor samples from 34 patients were analyzed by FISH for 1p36 deletion (left) and for UBE4B deletion (right). 7 tumor samples (21%) were found to have both 1p36 and UBE4B deletion (top images); all 27 other samples had neither 1p36 nor UBE4B deletion (bottom images).

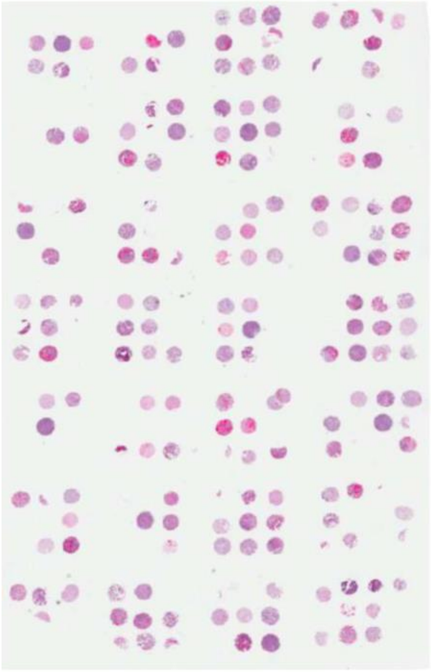

Neuroblastoma Tumor Tissue Microarray	
91 unique cases (6 control cases), each core 0.6mm in diameter	
Tissue types	number of cases
**Control Samples**	
Ganglioneuroma	3
Tonsil	3
**Neuroblastoma Samples**	
Stage 1	16
Stage 2	16
Stage 3	15
Stage 4	29
Stage 4S	15

Supplemental Figure 5: Neuroblastoma Tumor Tissue Microarrays. Neuroblastoma tumor tissue microarrays (TMAs) were obtained from COG. Photographs of the H&E stained TMA (left) and relevant information about tumor samples on the TMA (right) are shown.

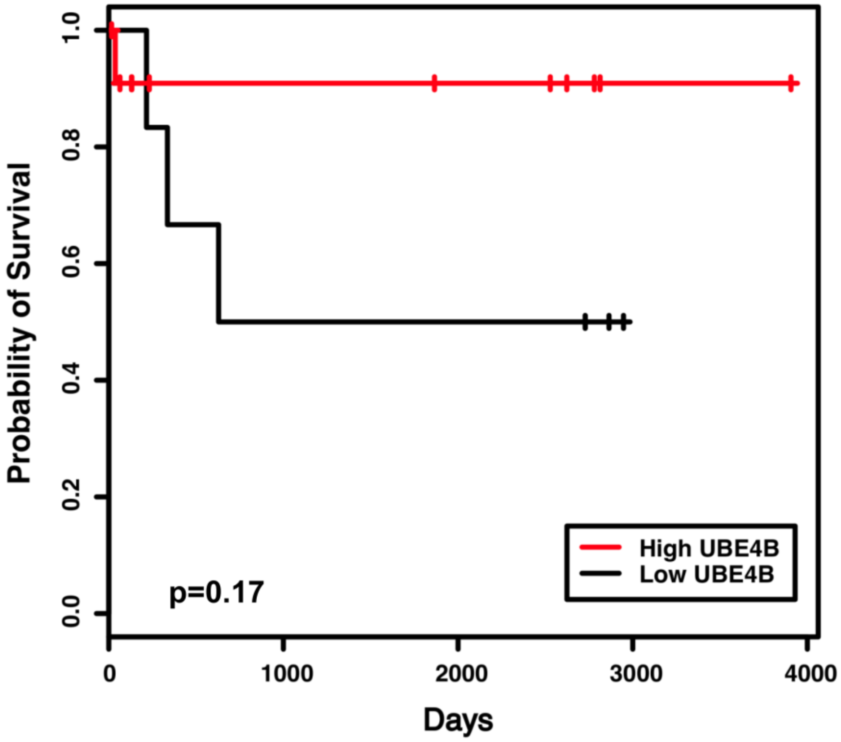

Supplemental Figure 6: Neuroblastoma Patient Survival Based on UBE4B Protein Expression in Neuroblastoma Tumor Tissue Arrays. Neuroblastoma tumor tissue microarrays (TMAs) were stained for UBE4B and Kaplan-Meier curves were generated for event-free survival for patients with low (n=7) and high (n=12) UBE4B protein expression.

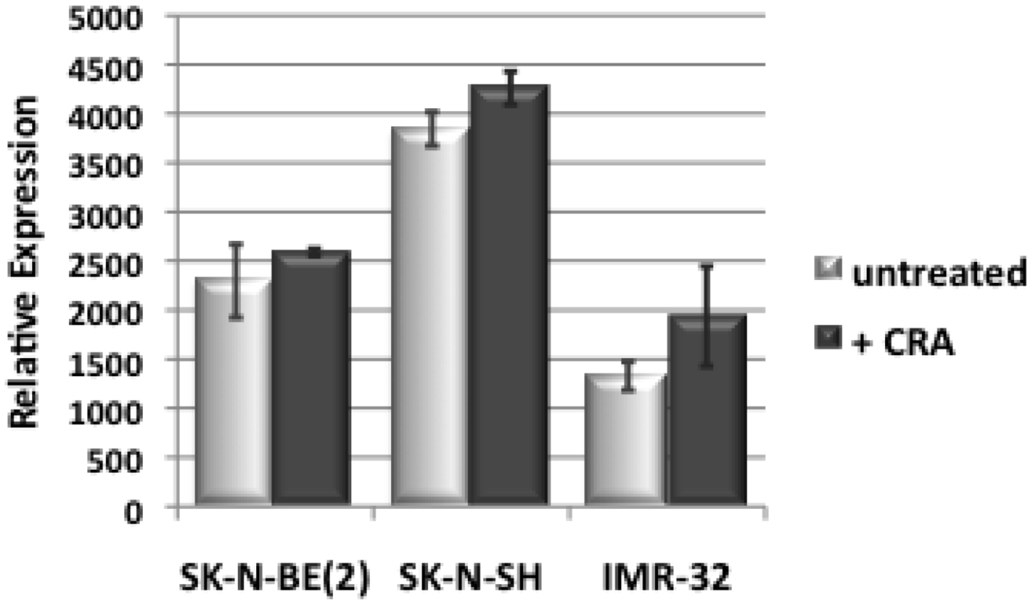

Supplemental Figure 7: UBE4B Gene Expression Before and After Retinoic Acid Treatment. Relative *UBE4B* gene expression in neuroblastoma tumor cell lines before ("untreated") and after ("+CRA") treatment with 13-*cis*-retinoic acid for 48 hours measured by RNA-sequencing is shown.
